# A case of parotid Acinic Cell Carcinoma in a young boy

**DOI:** 10.1590/S1808-86942011000300022

**Published:** 2015-10-19

**Authors:** Chiara Bianchini, Andrea Ciorba, Francesco Stomeo, Stefano Pelucchi, Massimo Pedriali, Antonio Pastore

**Affiliations:** 1 PhD (Clinical Assistant); 2 MD (ENT Department, University Hospital of Ferrara); 3 MD (ENT Department, University Hospital of Ferrara); 4 MD (ENT Department, University Hospital of Ferrara); 5 MD (Anatomic Pathology Section, University Hospital of Ferrara); 6 Prof (ENT Department, University Hospital of Ferrara); ENT Department and *Anatomic Pathology Section, University Hospital of Ferrara, Corso Giovecca 203, 44100 Ferrara, Italy

**Keywords:** carcinoma, child, parotid gland

## INTRODUCTION

Ephitelial salivary gland neoplasms are rare in children and adolescents: they represent only the 1%-5% of the total number of salivary gland tumours^1^.

In the infancy, it has been reported that 35% of salivary gland tumours are malignant and between these mucoepidermoid carcinoma is the most frequent, while the occurrence of acinic cell carcinoma in children is very rare^1^. The Authors are presenting a case of an acinic cell carcinoma of the parotid gland in a 15-year-old boy.

## CASE REPORT

A 15-year-old boy was referred to the ENT Department at the University Hospital of Ferrara, for the assessment of a right, painless, firm, preauricolar mass, which was noticed six months before. The ecographic evaluation revealed the presence of an irregular surfaced, 2.5 cm maximum diameter, right parotid mass and a 1 cm reactive lymphoadenopathy located at the inferior parotid edge. A fine needle aspiration cytology (FNAC) was performed and revealed a possible epithelial salivary gland tumour containing acinic cells.

Even if facial nerve function appeared normal, facial electromyography (EMG) was performed prior to surgery, and it proved a little action potential amplitude asimmetry in the inferior right area of the face.

A total parotidectomy with preservation of the facial nerve was performed, under general anesthesia. The mass was hard, poorly circumscribed, not encapsulated and situated both in the superficial and deep portion of the gland with intimal involvement of the superior branches of the right facial nerve.

**Fig 1:**
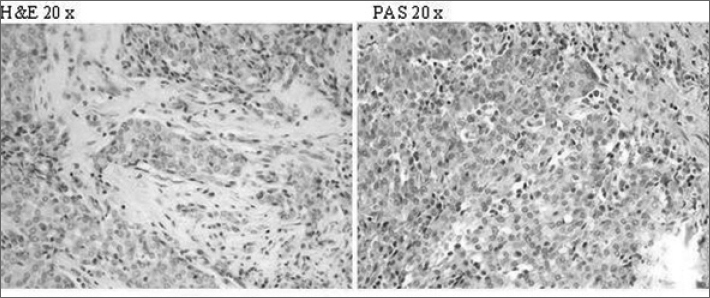
The microscopic appearance shows uniform cells with small central nucleus and abundant basophilic cytoplasm which can appear granular or vacuolated, in both stains (EE and PAS). Mucin stain was negative. The most characteristic cells have a cytoplasmic appearance, an ultrastructural morphology and a secretory behavior pattern analogous to those of acinic cells of normal salivary glands.

Histologic examination revealed an infiltrating Acinic Cells Carcinoma with no metastasis in the six lymphnodes found inside the specimen (fig. 1).

Only a mild weakness of right eye's orbicular muscle was noted in the first two postoperative days. The patient underwent a single cycle of radiotherapy (5400 cGy total dose).

After 5 years of follow up, the patient is still disease free.

## DISCUSSION

Malignant epithelial salivary gland neoplasms are infrequent in adults and rare in children, as there only are few reports in the literature. Published data suggest a male preponderance; it is stimated a 1.4:1 male/female ratio. The major salivary glands, the parotid and submandibular, are the main site of occurrence^1^, ^2^, ^3^, ^4^, ^5^, ^6^.

Mucoepidermoid carcinoma is the most common malignant salivary tumour of the childhood^2^ whereas Acinic cell carcinoma in children is very rare, since it represents about the 3% of all malignant parotid neoplasms^3^. According to their malignant potential, Acinic Cell Carcinomas have been divided into three grades. Grades I (low-grade malignancy) comprises completely encapsulated tumours without local infiltration; grade II (moderate malignancy) tumours show signs of capsular invasion; and grade III (high-grade malignancy) have papillary-cystic zones and infiltrate the surrounding tissues^1^. It has been reported that Acinic Cell Carcinoma may invade adjacent tissues, and in 5 up to 10% of cases metastasis to regional lymphnodes or to distant organs may be present^1^, ^3^. Factors related to a poor prognosis are tumour size, an increased histological grade, histological type, facial palsy at diagnosis, pain, local invasion, rapid tumour growth and the presence of regional or distant metastasis^1^.

The clinical evaluation of a parotid mass can be difficult in children^1^, ^3^. Clinically, these lesions manifest as a painless, enlarging lesions and grow slowly, without symptoms. Facial nerve involvement is unusual, although, when present, should be considered as a sign of malignancy^1^, ^3^. In the reported case, the patient presented a painless pre-auricular mass, gradually enlarging during the last six months without signs of facial nerve deficit.

Ultrasonografy can be helpful for studying tumours topography and dimension. Computed tomography (CT) and magnetic resonance imaging (MRI) are also useful providing additional information regarding the local extension^1^, ^3^.

Fine-needle aspiration cytology (FNAC) can be considered a safe, rapid and helpful diagnostic test in determining the nature of a parotid mass. As it has been reported to have a specificity of 91% and a sensivity of 96% when sufficient cells are present^4^, ^5^. Nevertheless, in the pediatric population, patient tolerance of this procedure can limit its use^4^.

Differential diagnosis includes neoplastic lesions, vascular malformations, acute and chronic cervical lymphadenopathies and cystic lesions^1^, ^2^, ^3^, ^4^, ^5^, ^6^.

Patient history, physical examination, radiological studies, all are necessary for treatment planning. Surgery is the treatment of choice for epithelial salivary gland tumours in both adults and children.

In children adjuvant radiotherapy should be considered only in selected cases for its possible complications; these include trismus, retarded growth of the facial bones, hemi-facial hypoplasia, pituitary insufficiency and X-ray induced cancer^1^, ^6^. Mainly indications for radiation therapy after surgery are residual disease, histological high grade lesions, soft-tissue invasion, cervical limph node metastasis, perineural facial nerve and vascular extension^1^, ^3^. Our case was treated due to the perineural involvement, as clinically evident at the EMG.

Our patient has shown no evidence of recurrence after 5 years of clinical and radiological follow-up.

## FINAL REMARKS

Malignant epithelial salivary gland neoplasms are infrequent in adults and very rare in children. We support the idea that the best treatment for parotid malignancies in children as in adult age is surgery and only in selected cases adjuvant radiation therapy is necessary.
